# Global evidence of persistent violations of the International Code of Marketing of Breast‐milk Substitutes: A systematic scoping review

**DOI:** 10.1111/mcn.13335

**Published:** 2022-03-21

**Authors:** Genevieve E. Becker, Paul Zambrano, Constance Ching, Jennifer Cashin, Allison Burns, Eva Policarpo, Janice Datu‐Sanguyo, Roger Mathisen

**Affiliations:** ^1^ BEST Services Galway City Ireland; ^2^ Alive & Thrive Southeast Asia/FHI 360 Manila Philippines; ^3^ Alive & Thrive Southeast Asia, FHI 360 Washington District of Columbia USA; ^4^ FHI 360 Durham North Carolina USA; ^5^ Independent Galway City Ireland

**Keywords:** breastfeeding, breastmilk substitutes, code of marketing, compliance, infant food, infant formula, scoping review

## Abstract

The influence of marketing on infant and young child feeding and health is well recognized, and an International Code was adopted by the World Health Assembly (WHA) in 1981 to reduce inappropriate marketing and protect breastfeeding. Yet the marketing and influencing continue. This scoping review systematically examined the published research evidence on the nature and extent of exposure to International Code violations from 1981 to August 2021. We used several search strategies involving multi‐language databases, organization websites, citation tracking, and expert consultation, to find research items meeting our inclusion criteria. We evaluated 657 items and retained 153 studies from at least 95 countries in the review. The majority of the studies (*n* = 113) documenting exposure to inappropriate marketing were published since 2010. Studies reported a broad range of marketing violations targeting mothers and families, health workers, and the general public. Marketing via digital platforms and brand extension has become more frequent. The evidence shows the use of misleading and inaccurate labeling and health and nutrition claims in breach of the Code. Our review confirms that violations of the Code have not ceased and calls for renewed attention from the WHA and national governments to protect the health of children and their mothers.

## Highlights

- Exposure to inappropriate marketing continues across the world in health systems, public spaces, points of sale, media, emergency programs, and direct to mothers.
- Studies have documented the emergence of products and marketing practices designed to circumvent the Code.
- Labeling and health and nutrition claims were found that were misleading, inaccurate, and in breach of the Code.
- Evidence of marketing through social media and other digital platforms has increased over time.
- Future research, guidance, and policies would benefit from an accessible central repository of comprehensive and comparable measurement tools and evidence.

## Graphical abstract

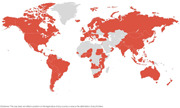

## 1 INTRODUCTION

Corporate efforts to weaken public health policies and influence research and practice to increase corporate profits are recognized across a range of products that contribute to ill‐health (Mialon, 2020; World Health Organization, 2013). Though the term “commercial determinants of health” may be recent (Kickbusch et al., 2016), awareness of the overall concept that marketing and other corporate strategies influence the knowledge, attitudes, and behaviors of pregnant women and new mothers, her family, and friends, the general public, health workers, and of policymakers is not new. Inappropriate marketing, namely, promotion, of breastmilk substitutes (BMS) by the baby food industry negatively impacts immediate and long‐term health outcomes for mothers and children as well as household and community socioeconomic status (Piwoz & Huffman, 2015; Rollins et al., 2016; Walters et al., 2019).

Recognition of the negative health effects of this marketing resulted in the adoption by the World Health Assembly (WHA) of the International Code of Marketing of Breast‐milk Substitutes (the Code) in 1981 (World Health Organization, 1981). Subsequent WHA Resolutions were passed to update the Code in the context of changing marketing practices and new scientific findings and recommendations on infant and young child feeding (IYCF). For example, recommendations on complementary feeding and continued breastfeeding brought forth by WHA Resolutions 54.2 (2001) and 58.32 (2005) led to the understanding that any milk product, food, and beverage marketed as a replacement for breastmilk during the period of six months to two years or beyond is subject to the Code. Health and nutrition claims are restricted by WHA Resolution 58.32 (2005), and WHA Resolution 63.23 (2010) calls on governments to ensure the prohibition of donations of BMS, complementary foods, and infant feeding equipment in emergencies. In 2016, the WHO Guidance on Ending the Inappropriate Promotion of Foods for Infants and Young Children (hereafter “the 2016 Guidance”) enshrined in WHA Resolution 69.9 not only clarifies that follow‐up formulas and growing‐up milk are BMS, in addition it provides recommendations aiming to end inappropriate promotion of commercial complementary foods for infants and young children from 6 months to 3 years, including safeguards to prevent conflicts of interest in health systems (IBFAN‐ICDC, 2018; Theurich, 2018).

The Code, together with subsequent relevant resolutions to date (hereafter collectively referred to as “the Code”), apply to all products that are marketed or represented as a suitable partial or total replacement of breastmilk, including any milk products marketed for feeding infants and young children up to 3 years of age, other foods and beverages marketed as suitable for feeding infants less than 6 months or for feeding on a bottle. The Code also applies to feeding bottles and teats, and complementary foods marketed for children up to 3 years of age. The Code calls on all Member States to ensure that effective, objective, independent, and transparent monitoring systems are in place to enforce the Code's standards and recommendations. It is also a part of global guidance and recommendations to reduce the impact of food marketing on child health (World Health Organization, 2020).

With the aim to protect breastfeeding and optimal IYCF, the Code prohibits the promotion of covered products (with the exception of commercial complementary foods of which only some forms of promotion are restricted), including promotion to the general public, health workers, and mothers; via media, provision of gifts and incentives to health workers and to mothers, use of health claims and cross‐promotion between complementary foods and BMS. Donations or low‐cost supplies of BMS to health services and in emergency situations, donated equipment to health care facilities, financial support for conferences, provision of education sessions, and other incentives are also prohibited, as these can all serve to build goodwill within the health system and with the public, increase the use and potentially expand the market of the products, while also creating conflicts of interest. Despite assertions by the industry that BMS companies are compliant with the Code (Nestlé, 2018) and commentary that marketing does not influence feeding practices (Bognar et al., 2020; Forsyth, 2013), numerous recent studies indicate that promotion through advertisement, gifts, sponsorship, cross‐promotion, and inappropriate labeling remains a problem with negative effects on infant feeding attitudes and behaviors (Berry et al., 2010; Boyle & Shamji, 2021; Ching et al., 2021; Hastings et al., 2020; Save the Children, 2018).

Various survey tools have been used by organizations to monitor the Code, the most commonly used being those developed by the Interagency Group on Breastfeeding Monitoring (IGBM, 1997) and more recently, NetCode (World Health Organization, 2017), with International Baby Foods Action Network (IBFAN) tools in use over the years (IBFAN‐ICDC, 2018), as well as tools devised for specific research studies. Despite the proliferation of published research related to the Code undertaken by a broad range of disciplines and foci, including health promotion, clinical care, sociology, marketing, gender, economics, environment, and others, a preliminary search for existing reviews of published research on exposure to violations of the Code indicated no existing reviews. Given the need to explore a broad body of evidence in a systematic manner to provide a concise knowledge synthesis that may aid future planning, a scoping review was identified as an appropriate method (Cochrane Training, undated).

Multiple actions are required to reduce the negative influence of corporate strategies on IYCF, including Code implementation and enforcement of national policies, monitoring of regulations, and reducing exposure to marketing. The aim of this independent scoping review was to systematically examine and summarise the published research evidence of exposure to violations of the Code worldwide, to identify the available research on the topic, examine the settings where exposure occurred, who was targeted, what the marketing activities were, and what products were involved.

## 2 METHODS

This systematic scoping review followed the Joanna Briggs Institute guidelines (Peters et al., 2020, chap. 11) and is reported in accordance with the Preferred Reporting Items for Systematic reviews and Meta‐Analysis (PRISMA) extension for Scoping Reviews (Tricco et al., 2018). The six‐step process of Arksey and O'Malley (Arksey & O'Malley, 2005) formed the framework and a protocol was published (Becker et al., 2021, 2019). The overall research question was identified as: *What is the published evidence of exposure to violations of the International Code of Marketing of Breast‐milk Substitutes*?

An international consultative group, including researchers and persons involved in guidance and monitoring of Code implementation, was consulted at the protocol stage to develop the research question, plan the search strategy, discuss data elements to be charted and key elements to report; then to review the list of Included and Excluded items after the screening phase to ensure no key documents were missed, and to comment on our findings before publication.

Search terms were developed from an analysis of keywords in known articles relevant to the Code, BMS, violation/compliance, and marketing. The search strategy was designed to be geographically broad and extend over four decades rather than aiming for saturation, and to provide the review in a timely manner.

Multiple sources were chosen including (i) multiple broad disciplinary bibliographic databases, (ii) websites of organizations identified as closely involved with the Code, (iii) review of the references and citations of included items, and (iv) additional items suggested by the consultation group. The PRISMA‐S checklist guided the search (Rethlefsen et al., 2021; Supporting Information A: Search strategy, databases, and organization websites searched).

Studies were considered with data collected or published from May 1981 (the date when the WHA adopted the Code) until July 2021. No limitations were set for geographical location, country income level, social or cultural group, language, setting in which the violation occurs, or journal impact factor. The authors of this study include proficient speakers of Chinese, Spanish, French, and Portuguese as well as English. Documents in languages other than these five languages for which the abstract could be translated adequately using Google translate were included for screening with the full text translated as needed. Studies were considered from settings including but not limited to retail, health care, emergency or relief settings and/or directed at any group (pregnant women, new mothers, health workers, and general public), and/or via any means (leaflets, digital media, free samples, etc.).

A screening tool was developed and tested, and abstracts were independently screened by two members of the team. Eligibility for inclusion required that all three criteria were met:

1. Document is a primary report of a systematic investigation, including a research question or problem, method of enquiry, analysis method, and reporting of findings.
2. Document reports on specific violation(s) of the Code.
3. Document reports on one or more specific context(s), setting(s), or means of marketing.

Opinion papers, policies, guidelines, reviews, and studies solely focused on the effects of violations were excluded. Multiple reports of the same study were combined as one item of evidence. Pacifiers and commercial milk formula for pregnant women or lactating women are not currently within the scope of the Code however they are an increasing means of cross‐promotion, undermining breastfeeding, and therefore we included them in this review.

A data charting tool for eligible studies was prepared using headings determined at the protocol stage (Supporting Information Appendix A: Charting headings) to answer key research aspects:

- What types of published research examined Code violations?
- When were the studies conducted?
- How geographically widespread were the studies?
- In which settings did the violations occur?
- Who were the targets of violations?
- What are the types of violations reported?
- What products were involved in the violations?

The charting tool was piloted, and five studies were charted together to develop consistency. Team members independently reviewed their assigned studies with all items second charted by another team member to facilitate accuracy, with any disagreements discussed between the two team members.

The spreadsheet charting tool was used for the basic numerical analysis and summarised in tabular format with simple descriptive statistics as relevant. The studies are described, and findings are reported as a thematic narrative summary linked to the seven aspects of the research question.

## 3 RESULTS

**Figure 1:**
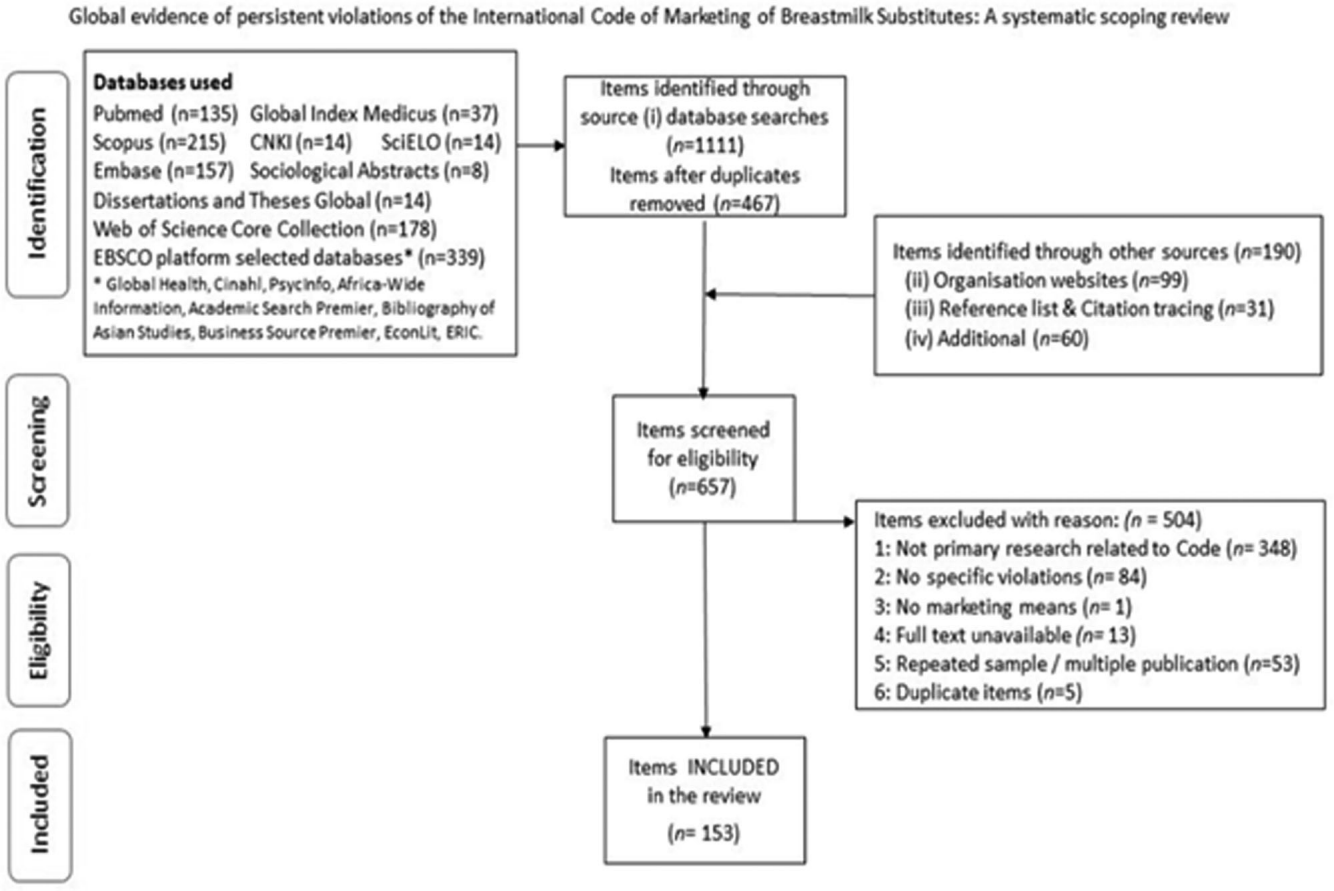
Search results

The four search sources identified 1301 items, which after the electronic database de‐duplication process resulted in 657 items to be screened for eligibility. The screening process resulted in 153 studies eligible for charting (data extraction; Figure 1; Supporting Information Appendix B: List of eligible studies).

### 3.1 Types of published research that examined Code violations

Table 1 summarises the studies included in our review published from the adoption of the Code in 1981 to mid‐2021.

**Table 1:** Summary of studies

	Number of studies
*Publication type (total n = 153)*	
Journal	91
Organisation report	41
Academic thesis	13
Conference abstract	5
Other	3
*Source of funding (multiple funding sources could be reported)*	
Philanthropic foundation	32
UN agency	23
Government	18
University or research institute	12
Nongovernmental organization (NGO)	11
Self‐funded	3
IYCF company	2
Other	13
Not specified or stated none	56
*Data collection method (some studies used multiple methods)*	
Observational tool, checklist	84
Interview/questionnaire	75
Media scan/Internet search	58
Documents and records	26
Other (focus groups, laboratory analysis)	5
*Used (or adapted) a pre‐existing survey tool (n = 78)*	
IBFAN	24
NetCode	13
IGBM	9
Other (mixed)	32
*Studies with this sample type n (sample size range; some studies included multiple sample types)*	
Pregnant women/mothers/caregivers	61 (6–6102)
Health workers	38 (8–669)
Points of sale	49 (4–399)
Health facilities	45 (2–1239)
Products/labels	54 (1–978)
TV and radio channels	27 (2–270)
Websites, social media and other digital	33 (1–400)
Magazines, newspapers, journals (print)	33 (1–207)
Informational materials	11 (1–22)
Examples of violations/practices	12 (1–1280)
Brands/companies	17 (4–101)
Other (people of other types, cities/countries, events, schools)	11 (n/a)
*Decade of study (total n = 153) date of data collection or if not stated, the date of publication. If a range crosses a decade, then the mid‐point of the range is used*	
1981–1989	2
1990–1999	12
2000–2009	26
2010–2019	99
2020–August 2021	14

Abbreviations: IBFAN, International Baby Foods Action Network; IGBM, Interagency Group on Breastfeeding Monitoring; IYCF, infant and young child feeding; TV, television.

Most studies were published in peer‐review journals (59%; *n* = 91) in various formats. When a funding source was disclosed, it was most commonly a philanthropic foundation (*n* = 32). No study disclosed major funding from a company producing BMS; two studies noted funding in the form of an individual research grant from the industry to one of the researchers (Mialon et al., 2021) and part‐funding of another study (Popkin et al., 1990); these two studies were included in the review.

A variety of data collection methods were used to document Code violations. An observation tool or checklist was the most common method of data collection with three global pre‐existing tools used in 59% of the studies that used survey tools. Pregnant women and/or mothers were the most sampled population for interviews. Study size ranged from examination of a single product or information source and small studies using qualitative interviews to very large quantitative studies.

### 3.2 When the studies of violations were conducted

Research on Code violations has increased dramatically over time, with 99 studies published from 2010 to 2019 and 14 studies published in the 18 months from January 2020 to July 2021. For the purposes of analysis, the period from January 2020 to July 2021 was combined with the period from 2010 to 2019.

### 3.3 Geographic spread of the studies

**Figure 2:**
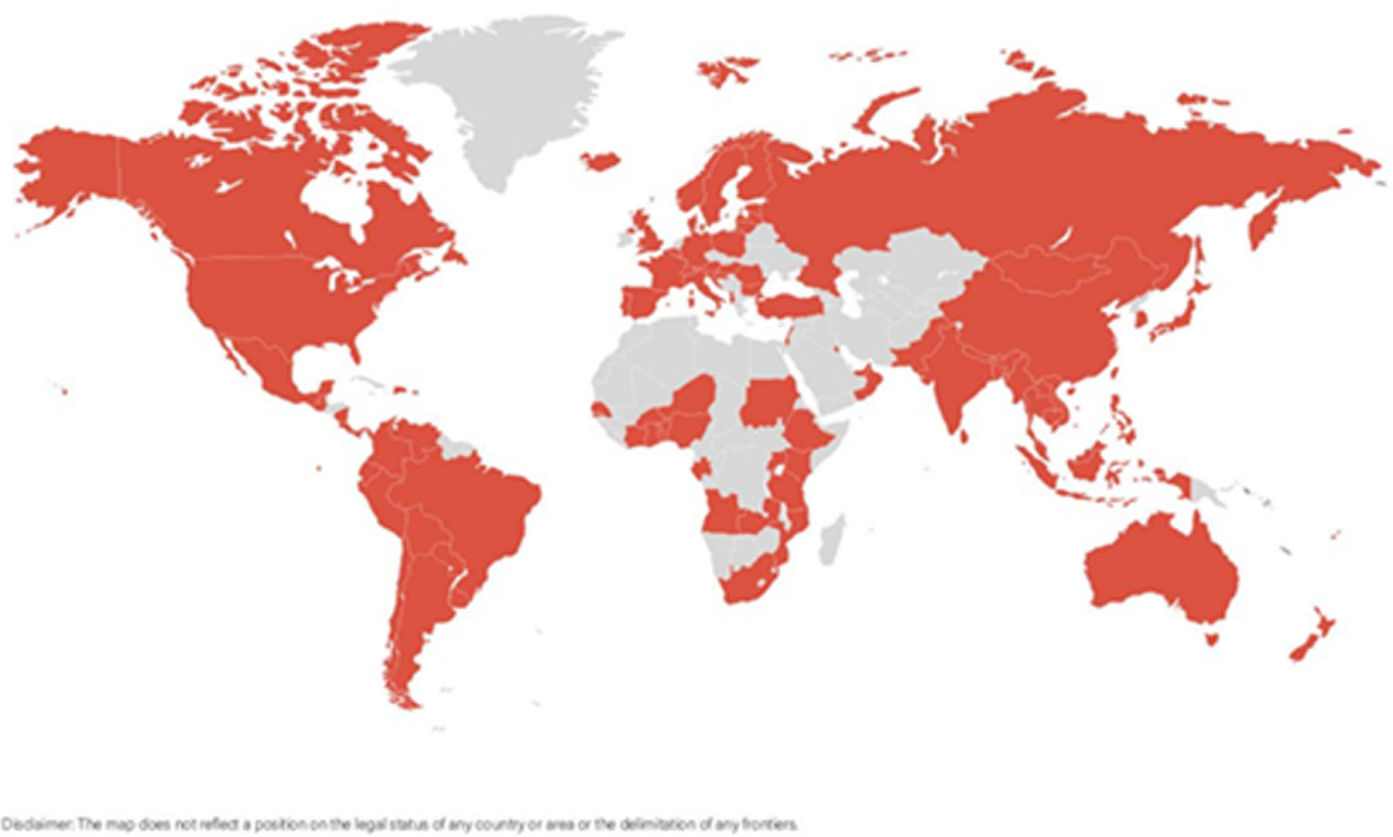
Countries with at least one study documenting a violation of the International Code of Marketing of Breast‐milk Substitutes.

Studies documenting Code violations were widespread and found in at least 95 countries (Figure 2). Eight studies reported violations at a regional or global level without naming the individual countries (Supporting Information Appendix B: List of countries). Some studies involved more than one country and each named country with data is listed. Evidence of Code violations was found in all WHO regions.

### 3.4 Settings where the violations occurred

**Figure 3:**
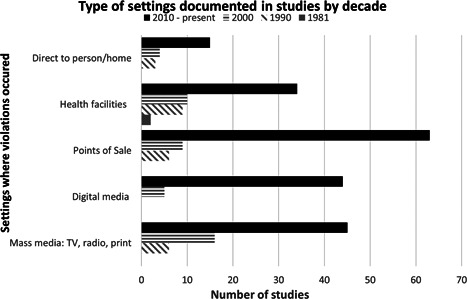
Types of settings where violations took place, documented in studies by decade

Code violations occurred in a variety of settings. Point of sale violations (physical and online retailers) were most documented (*n* = 78), followed by violations in mass media (such as TV, radio, and print; *n* = 67), and health facilities (*n* = 55; Figure 3). Studies involving violations on digital media were found only from the year 2000 but have undergone a sharp increase since then (from *n* = 5 to 44), corresponding to the evolving digital marketing landscape. There was also an increase over time in studies reporting direct‐to‐person marketing, including invitations for potential consumers to contact the manufacturer, which resulted in samples being mailed directly to homes.

The studies also reported other settings where Code violations occurred, including emergency programs and government and NGO programs (*n* = 13), public spaces (such as billboards on roads and public transport; *n* = 12), health workers' training and education (*n* = 10) and daycare facilities and schools (*n* = 5). Some studies examined more than one setting and each setting is counted separately.

### 3.5 Targets of inappropriate marketing

**Figure 4:**
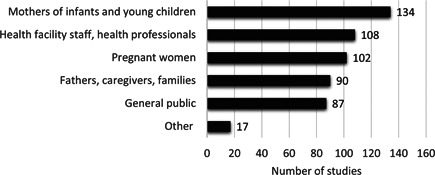
Targets of inappropriate marketing of breastmilk substitutes

Mothers of infants and young children were the most frequently identified target of BMS marketing (134 studies, 87.5%; Figure 4). Health facility staff, health professionals (including students), and professional associations were the next most common target followed by pregnant women, then fathers, caregivers and families, and the general public. Other targets included retail staff in pharmacies and shops, policymakers and government officials, school children, and researchers. Violations targeting mothers, pregnant women, health workers, and fathers, caregivers, and families were identified in studies across all decades. Many studies in our sample reported marketing across multiple target groups and we counted each target group separately.

### 3.6 Types of Code violations reported in studies

The wide range of Code violations reported in the studies was categorized into types broadly based on the relevant Articles (Art.) in the Code and WHA resolutions (Res.). Some studies reported more than one type of violation and each type is counted separately.

As shown in Table 2, marketing practices related to advertisement and promotion on other platforms (such as those in print and on social media) were the most common type of violations reported in the studies (*n* = 96), followed by violations related to labeling (*n* = 86), giving samples and gifts (*n* = 77) and promotion in shops (*n* = 69). Promotion via health workers (*n* = 67) and the health system (*n* = 64), as well as violations through nutrition and health claims (n = 66), were also among the most frequently reported. Similar to violations through labeling and claims, studies documenting cross‐promotion (*n* = 54) emerged in the 1990s and increased in frequency over time. The main cross‐promotion categories documented were across BMS products (specifically between infant formula and other BMS products, such as follow‐up formula and growing‐up milk), and indirect promotion of BMS through cross‐branding with complementary foods, which undermines breastfeeding and is clarified as a violation in WHA Resolution 69.9 in 2016.

**Table 2:** Types of violations documented in studies, by decade

	Number of studies examining this aspect				
	1981–1989 (*N* = 2)	1990–1999 (*N* = 12)	2000–2009 (*N* = 26)	2010–Aug 2021 (*N* = 113)	Total 1981–Aug 2021 (*N* = 153)
*Types of violations*					
Provision of information and education (Art. 4)					
Public	1	7	7	44	*59*
Health workers	0	5	6	21	*32*
Promotion to general public and mothers (Art. 5)					
Shops	0	5	8	56	*69*
Advertisement and promotion on other platforms	1	8	16	71	*96*
Free sample and gifts	1	11	10	55	*77*
Contact with women (Art. 8)	1	3	7	38	*49*
Marketing activities in health care settings and through health workers (Art. 6 and 7)					
Health care systems	2	8	13	41	*64*
Health workers	0	11	12	44	*67*
Free or low‐cost supplies	0	6	8	20	*34*
Conflicts of interest (Res. 49.15 and 58.32)	1	3	5	12	*21*
Violations in labelling and packaging (Art. 9 and 10)					
Labelling	0	6	12	68	*86*
Nutrition and health claims (Res. 58.32 and 63.23)	0	5	7	54	*66*
Inappropriate marketing concerning CF (Res. 69.9)					
Inappropriate messaging and conflicts of interest in health systems	0	5	4	28	*37*
Cross‐promoting products					
Cross‐promotion across BMS and with CF (Res. 69.9)	0	5	6	43	*54*

Abbreviations: BMS, breastmilk substitutes; CF, complementary foods.

The main types of violations that studies reported varied across countries. For example, 41% of interviewed mothers in a study from Ethiopia reported that they had seen BMS advertising, and 74% of the violations reported occurring through TV promotions (Laillou et al., 2021). Promotion via parent magazines was highlighted in a study in Taiwan (Chen) and in a Chinese study “premiumization” and “nutrition and science” were the main marketing appeal strategies on the BMS companies' websites and e‐commerce platforms studied (Han, 2020). Free samples of formula soon after birth were a common type of violation studied in the USA (Sadacharan et al., 2011).

Eighty‐six studies reported violations related to labeling and packaging. Some examples included a study examining nine companies in 14 countries (Burkina Faso, Canada, China, India, Indonesia, Kenya, Laos, Malaysia, Myanmar, Pakistan, Singapore, The Philippines, United States, and Vietnam; Ching et al., 2021) and another carried out in four countries (Cambodia, Nepal, Senegal, and Tanzania; Sweet et al., 2016), which found explicit invitations on the packaging to interact with the manufacturer's “nutrition experts” or join a baby club for special offers (Ching et al., 2021; Sweet et al., 2016). Similar branding used across products for different ages was found in Nepal (Pries et al., 2016), and a lack of information about risks from improper use was reported in Uruguay (Alcaire et al., 2020). In a study in Ecuador, more than half of the BMS labels contained texts or images that idealized their use (Caicedo‐Borrás et al., 2021). Promotion via nutrition and health claims was reported in 66 studies, including statements making claims to benefit child health and development from Cambodia and Nepal (Champeny et al., 2019) and from a multi‐country study in Europe (Austria, Bulgaria, Hungary, and Israel; WHO Regional Office for Europe, 2019a), to help with digestion, support for cognitive development and aid to the immune system in an Australian study (Berry & Gribble, 2017). Promotion of feeding bottles and teats also included claims such as “proven to reduce colic” reported in Uruguay (Alcaire et al., 2020).

Marketing activities in health care settings and through health workers were numerous with many studies reporting more than one type of Code violation. For example, marketing activities related to scholarships, sponsorship of conferences and other events, or using a health facility to hold a marketing event were reported in country‐specific studies from Chile (Achurra & Salinas, 1993; Departamento de Nutrición, F. d. M., Universidad de Chile & PAHO, 2017), Ecuador (Bertha & Caicedo‐Borrás, 2016; Caicedo‐Borrás et al., 2021), Curacao (Kharade, 2019), Brazil (Rea & Toma, 2000) and Uruguay (Ministerio de Salud Organización Panamericana de la Salud & UNICEF, 2019), plus a multi‐country study from Latin America and South America (Mialon et al., 2021); and country‐specific studies from South Africa (Muravha, 2014), Uganda (IGBM et al., 2005; Ministry of Health UNICEF & IBFAN Uganda, 2011), Nigeria (Brewer et al., 2018), Cote d'Ivoire (Emerson et al., 2021); as well as reported in studies from Thailand (Cetthakrikul et al., 2014), Indonesia (Asosisasi Ibu Menyusui Indonesia [AIMI], 2021), Philippines (B. K. Brewer et al., 2021), Vietnam (Nguyen et al., 2021), Pakistan (Salasibew et al., 2008) and India (Gupta, 2021). These marketing activities were also found in studies carried out in 14 countries (Ching et al., 2021), 5 continents (Grummer‐Strawn et al., 2019), 3 continents (Hastings et al., 2020), and in many countries included in the Breaking the Rules reports (Yeong & Allain, 2001, 2004).

### 3.7 Products involved in the studies of Code violations

Many studies identified more than one product being marketed and we counted these separately. Infant formula (including formula marketed as “specialized”) comprised the largest proportion of all products documented across all decades, though showing a decreasing trend. We found an increase in the proportion of studies reporting inappropriate marketing of follow‐up formula and “growing‐up” or “toddler” milk from one study and no studies before 1990 to 59% and 53%, respectively, during the most recent decade (Table 3).

**Table 3:** Products documented as marketing violations, number of studies by decade

	Number of studies per decade (% of decade's studies)				
Product category	1981–1989 (*N* = 2)	1990–1999 (*N* = 12)	2000–2009 (*N* = 26)	2010–2021 (*N* = 113)	Total (*N* = 153)
Infant formula, including “specialised” formula	2 (100)	12 (100)	22 (88)	88 (77)	124 (81)
Follow‐up formula	1 (50)	4 (33)	9 (35)	67 (59)	81 (53)
“Growing‐up” or “toddler” milk	0 (0)	3 (35)	6 (23)	60 (53)	69 (45)
Breastmilk substitute, not specified	0 (0)	1 (8)	4 (15)	11 (10)	16 (10)
Other food/beverage marketed for infants <6 months or bottle feeding	0 (0)	4 (33)	4 (15)	27 (24)	35 (23)
Feeding bottles and teats	1 (50)	6 (50)	8 (31)	41 (37)	56 (37)
Complementary foods for children 6–36 months	0 (0)	4 (33)	9 (35)	45 (40)	58 (38)
Formula for pregnant and lactating women	0 (0)	2 (17)	3 (12)	7 (6)	12 (8)
Other products	2 (100)	1 (8)	6 (23)	19 (17)	28 (18)

*Note*: Some studies included more than one product and we counted each product separately.

Some studies used the generic term “breastmilk substitutes” with no specific information about the product(s) studied. Marketing of products other than infant formula for use before 6 months included juices and teas as well as cereal‐based products. Other products marketed specifically for infants included bottled water, teas, snack foods, fresh and soured milks, plant milks, and sweetener to use in a feeding bottle, which would be within the scope of the Code. Studies documenting the promotion of feeding bottles and teats were found in all decades. Studies also reported cross‐promotion of pacifiers, breast pumps, and feeding‐related equipment other than bottles and teats covered in national legislation but outside the scope of the International Code, with BMS. It is important to note that although commercial milk formula for pregnant and lactating women (CMF‐PW) is not within the scope of the Code, an increasing number of studies on Code violations reported on their inappropriate marketing that undermines breastfeeding, in particular cross‐branding strategies that indirectly promote BMSs (Nguyen et al., 2021).

## 4 DISCUSSION

This systematic review identified published studies of exposure to Code violations in at least 95 countries and all WHO regions. ALTHOUGH more countries have adopted national measures to give effect to some or all aspects of the Code over the years, and many believe that violations are no longer as prevalent as in the past, our review suggests that violations of the Code persist through an increasing range of marketing tactics.

The review reveals that the number of published reports of Code violations has increased substantially over time (from *n* = 2 in 1981–1989 to *n* = 153 in 2010–August 2021), possibly reflecting both increased adoption of the Code and interest in studying compliance and enforcement. Improvements in technical guidance for Code monitoring, data collection using digital tools, and increased investments in Code monitoring may have also contributed to this increase. The decade beginning 2020 includes only 18 months of studies and despite this short interval, 14 studies were found in our search, indicating continuing research activity. Some studies highlighted that observed marketing activities violating the Code appeared to increase during the COVID‐19 pandemic (AIMI, 2021; Ching et al., 2021), however, containment and prevention‐related restrictions may also reduce the number of studies involving interviews in recent and coming years (B. Brewer et al., 2021).

Our findings show that mothers and pregnant women continue to be among the most exposed to inappropriate marketing of products within the scope of the Code, but there is also substantial exposure among fathers, caregivers, and families. Marketing targeting the general public, including school children, help establish brand awareness, to normalize the use of BMS and commercially prepared complementary foods as well as to increase social acceptance of these products.

Support from health workers and within the health system is a well‐established determinant of IYCF practices (Matvienko‐Sikar et al., 2019; McFadden et al., 2017; Pries et al., 2016). As a trusted source of health advice to parents, health workers are an important target group for marketing, and health facilities are a long‐standing and major setting for the promotion of BMS (Ching et al., 2021; Nguyen et al., 2021; Rothstein et al., 2020; World Health Organization et al., 2020). Engaging the medical establishment also results in promotion by association (Hastings et al., 2020). Studies documented gifts, sponsorships, and other financial incentives to health workers that create conflicts of interest and compromise the integrity of information and advice given to parents (Pries et al., 2016; Rothstein et al., 2020). An exploratory survey and focus group analysis in the Philippines found that physicians' recommendations and self‐reported exposure to advertising were strongly associated with formula use (Sobel et al., 2011).

Retail is a major setting for BMS promotion and there is no mention in the Code of restrictions on marketing activities by companies to retail staff. Studies reported BMS companies training retail staff, including staff in pharmacies, on how to market their products and offering these staff sales incentives (Baby Milk Action UK, 2017; Oliveira et al., 2021).

Online purchasing of IYCF products is becoming more common and provides an opportunity for advertising, special offers, and other marketing practices via these retail sites. Social networking sites sponsored by BMS companies and the use of paid bloggers or influencers in recent years (Abrahams, 2012; Senkal & Yildiz, 2019) make digital marketing interactive, personalized, and thus effective. It is less apparent but more insidious as a form of commercial marketing to pregnant women and parents as well as policymakers. WHA Resolution 69.9 (2016) recognized the increasing diversity of communication channels. The increase in the number of studies examining and documenting violations on digital media platforms suggests a growing awareness of the risks posed by marketing on these platforms and the need for greater focus on WHA resolutions.

At the same time, inappropriate promotion persists in traditional media channels (TV and print) even in countries with national measures implemented which has resulted in calls for updating and strengthening of regulations (Vinje et al., 2017).

Studies highlighted the contribution of inappropriate promotion and messaging of complementary foods to the displacement of breastfeeding while also increasing the risks of both undernutrition and overnutrition (Sweet et al., 2016; WHO Regional Office for Europe, 2019b). While the Code does not regulate the composition of products, this type of violation is problematic especially with evidence of high sugar content in milk drinks marketed for 1–3‐year olds and complementary foods sweetened with concentrated fruit juice and fruit puree while also making the misleading claim of “no added sugar” (Bridge et al., 2020, 2021).

The increased popularity of online shopping portals and social media may have contributed to the increase in the number of studies documenting labeling violations, specifically violations regarding nutrition and health claims, as pack shots of products are often featured on these platforms. The two categories often co‐exist, where unsubstantiated and misleading claims systemically become the primary promotional and premiumization device.

Feeding young infants with unsuitable products puts their health at risk. Studies reported that caregivers mistook labels and advertisements of products such as follow‐on milk (marketed as suitable for 6–12‐month olds) and “toddler” milk as formula suitable for young infants (Berry et al., 2010; Cattaneo et al., 2015). One large study in a low‐income country found the same brand logo of a mother and baby bear used for both a sugar‐based coffee creamer and a milk product for older babies, which confused parents that the creamer was also suitable for infant feeding (Barennes et al., 2008).

Feeding bottles and teats continue to be marketed in a manner that undermines breastfeeding, and insufficient label warnings and safety instructions create potential health risks for infants using these products.

Our findings are consistent with the product development and subsequent differentiation of BMS since the adoption of the Code in 1981, especially the emergence of “growing‐up” or “toddler” milk marketed as suitable for children from 12 to 36 months, and the marketing of processed complementary foods under the same branding as BMS. This differentiation is one means of circumventing the Code to indirectly promote infant formula by marketing the other products with the same branding (Baker et al., 2021; World Health Organization & UNICEF, 2019). Studies documenting cross‐promotion within BMS products and between BMS and complementary foods were among the categories of violations that increased from 2010 in varied contexts, including Australia, India, Indonesia, Italy, the United Kingdom, and Vietnam.

We found evidence of inappropriate marketing of products currently not covered by the scope of the Code. Though teats are within the scope of the Code, pacifiers are not. Pacifier use has been linked to the disruption of exclusive breastfeeding (Buccini et al., 2017). The same company branding may be used for marketing the pacifiers thus cross‐promoting the teats. Marketing of pacifiers was documented in countries where they are covered by the national law, such as Brazil (Bartolini et al., 2009; Lima, 2019; Lopes & Pereira, 2013; Rodrigues et al., 2021) and Vietnam (Durako et al., 2016). Marketing of CMF‐PW can be a channel for cross‐promotion to circumvent the Code (Nguyen et al., 2021; Zhao et al., 2021, 2019). Pacifiers and commercial milk formulas for mothers should be included in future guidance on the Code and countries should be encouraged to implement measures to restrict cross‐promotion through the marketing of these products.

More than half of the studies (59%) that utilized a survey or monitoring tool using one of the three pre‐existing Code‐specific tools (IGBM, NetCode, and IBFAN). The NetCode tools (World Health Organization, 2017) were designed to guide national Code monitoring programs and may facilitate comparison of exposure to marketing violations between countries over the years. The Code and WHA resolutions call for the Member States to regularly report on their implementation, yet any national monitoring reports that we included in this review were obtained from multiple sources, not collected through a centralized system or repository. Such a repository could support efforts to sustain regular global reporting on the Code by the WHO Member States, facilitating reporting to WHA and other relevant advisory or decision‐making bodies, eliminating publication costs for underfunded researchers and organizations, as well as aiding standardization of methods for comparability.

Our review focused on the exposure of mothers, health workers, retail staff, and other individuals to marketing. However, marketing is multi‐faceted and aims to influence attitudes and behaviors. A person's understanding or perception of the products and the marketing may be as important as its occurrence and warrants review. Mothers (and other caregivers) have indicated confusion between BMS products being marketed (Barennes et al., 2008; Berry et al., 2010; Cattaneo et al., 2015) and health service and BMS industry interactions may be so normalized that the effect is not recognized (Bognar et al., 2020). It would be valuable to also collate the global research evidence on the effect of inappropriate marketing on family and national economics and environmental impact (Dadhich et al., 2021; Long et al., 2021; Smith, 2015; Walters et al., 2019) towards building the knowledge and developing coordinated multi‐faceted solutions.

### 4.1 Limitations

Our search may not have captured studies not accessible in online databases or that were published in languages not commonly found in major search databases, or from researchers without the resources for publication. This was mitigated by our search of multiple databases, no search restrictions to languages, reference checks, and through consultation with the international consultative group. However, it is not an exhaustive documentation of studies examining Code violations. Our focus was the research evidence of exposure to violations of the Code and did not review national legislation implementing and enforcing the Code.

## 5 CONCLUSION

Our systematic scoping review shows that four decades from the adoption of the Code by the WHA in 1981, well‐established and systemic forms of inappropriate marketing of BMS persist globally in violation of the Code. Evidence of emergent forms of Code violations involving digital marketing and more differentiated types of BMS validate the importance of regular reports to the WHA and adoption of subsequent resolutions and guidance to address gaps that may be used in the commercial marketing of BMS and associated products. Future studies would benefit from a centralized and open‐access database of materials relevant to Code violations. The findings of this systematic scoping review and their dissemination through open‐access publication and social media will contribute to further discussion and activity on the topic, serve to inform international and national decision‐makers, provide helpful insight to strengthen Code implementation, monitoring, and enforcement, and thus contribute to protecting the health of infants and young children and their mothers.

## CONFLICTS OF INTEREST

The authors declare that there are no conflicts of interest.

## AUTHOR CONTRIBUTIONS

Genevieve E. Becker led the development of the protocol, reporting the methods of the review, and overall coordination for writing the paper; Allison Burns carried out database searching and with Genevieve E. Becker reporting search results, searches of other sources were carried out by all team members. Constance Ching and Jennifer Cashin developed the data charting process; Janice Datu‐Sanguyo, Eva Policarpo, Jennifer Cashin, Constance Ching, Paul Zambrano, and Genevieve E. Becker carried out screening and charting of studies with Roger Mathisen also screening studies. Constance Ching and Paul Zambrano lead on the analysis of the results; project coordination was done by Genevieve E. Becker and Paul Zambrano; funding acquisition was coordinated by Roger Mathisen and Paul Zambrano. All authors participated in discussions, reviewed, and agreed to the published version of the manuscript.

## Supporting information

Supporting information.

Supporting information.

Supporting information.

Supporting information.

## Data Availability

As this article is a systematic review of existing data and no new data were collected, data sharing is not applicable to this article.
