# Supplementary material for: Global evidence of persistent violations of the International Code of Marketing of Breast‐milk Substitutes: A systematic scoping review
Source: Matern Child Nutr. 2022 Mar 21;18(Suppl 3):e13335. doi: 10.1111/mcn.13335 (PMC9113471; doi:10.1111/mcn.13335)
Supplement: Supplementary file 1 — Supporting information. [file MCN-18-e13335-s004.docx]

**Appendix A: Charting headings**

| Person Charting: | Date Charted: |
| --- | --- |
| Citation details (e.g., author/s, date, title, journal, volume, issue, pages) | |
| SECTION A: STUDY DESIGN AND INFORMATION | |
| 1. Where study is published (e.g., name of journal, organisation, etc.) | |
| 1. Date of publication | |
| 1. Source(s) of funding for the study | |
| 1. Publication type | |
| 1. Type of study and design | |
| 1. Data collection method | |
| 1. Data collection period | |
| 1. Use of pre-existing survey tool | |
| 1. Sample size (e.g., number of participants, sites, etc.) | |
| 1. Sampling method | |
| 1. Sample characteristics | |
| 1. Geographical location: Region/Country (or Countries)/City/Other (Specify) | |
| SECTION B: PRODUCTS AND VIOLATION | |
| 1. Product(s) being marketed | |
| 1. Types of violations | |
| 1. When violations happened/were documented | |
| 1. Where marketing is occurring | |
| 1. Who marketing is directed at | |
| 1. How marketing is carried out | |
| 1. Companies (and/or brands) reported in the study | |
| 1. Details of costs of the marketing | |
| SECTION C: OTHER INFORMATION | |
| 1. Further information needed from the study authors | |
| 1. References noted for potential inclusion or background | |
| 1. Additional details and notes | |
