# Supplementary material for: Global evidence of persistent violations of the International Code of Marketing of Breast‐milk Substitutes: A systematic scoping review
Source: Matern Child Nutr. 2022 Mar 21;18(Suppl 3):e13335. doi: 10.1111/mcn.13335 (PMC9113471; doi:10.1111/mcn.13335)
Supplement: Supplementary file 2 — Supporting information. [file MCN-18-e13335-s003.docx]

***Appendix B: List of eligible studies***

Abrahams S. W. (2012). Milk and social media: online communities and the International Code of Marketing of Breast-milk Substitutes. *Journal of Human Lactation*, 28(3), 400–406. https://doi.org/10.1177/0890334412447080

Access to Nutrition Initiative. (2021). *Landscape study: The Philippines, complementary feeding and the role of commercially produced complementary foods in young children's diets*. https://accesstonutrition.org/app/uploads/2021/05/ATNI_PH-CPCF-landscape-study.pdf

Achurra, X. & Salinas, J. (1993). Monitoring of the level of compliance with the conditions of the International Code of Marketing of Human Milk Substitutes. Revista Chilena de Nutrición. 21: 98-111.

Aguayo, V. M., Ross, J. S., Kanon, S., & Ouedraogo, A. N. (2003). Monitoring compliance with the International Code of Marketing of Breastmilk Substitutes in west Africa: multisite cross sectional survey in Togo and Burkina Faso. *BMJ* (Clinical research ed.), 326(7381), 127. https://doi.org/10.1136/bmj.326.7381.127

Al-Ghannami, S., Al-Shammakhi, S., Al Jawaldeh, A., Al-Mamari, F., Al Gammaria, I., Al-Aamry, J., & Mabry, R. (2019). Rapid assessment of marketing of unhealthy foods to children in mass media, schools and retail stores in Oman. *Eastern Mediterranean health journal*, 25(11), 820–827. https://doi.org/10.26719/emhj.19.066

Alcaire, F., Antúnez, L., Vidal, L., de León, C., Girona, A., Rodríguez, R., Giménez, A., Bove, I., & Ares, G. (2021). The idealisation of bottle feeding: content analysis of feeding bottles and teats packages in Uruguay. *Public health nutrition*, 24(10), 3147–3155. https://doi.org/10.1017/S1368980020004176

Aryeetey, R. N., & Tay, M. (2015). Compliance Audit of Processed Complementary Foods in Urban Ghana. *Frontiers in public health*, 3, 243. https://doi.org/10.3389/fpubh.2015.00243

Aryeetey, R.N. & Antwi, C. L. (2013). Re-assessment of selected Baby-Friendly maternity facilities in Accra, Ghana. *International breastfeeding journal*, 8(1), 15. https://doi.org/10.1186/1746-4358-8-15

Asosisasi Ibu Menyusui Indonesia (AIMI). (2021). *Breaking the Code: International Code Violations on Digital Platforms and Social Media in Indonesia during the COVID-19 Pandemic (April 2020-April 2021).* AIMI. http://www.babymilkaction.org/wp-content/uploads/2021/06/Breaking-the-Code-AIMI-Final.pdf

Baby Milk Action UK. (2017). *Look What They’re Doing 2017: How marketing of feeding products for infants and young children in the UK breaks the rules.* Baby Milk Action. http://www.babymilkaction.org/wp-content/uploads/2017/03/lwtduk17introduction.pdf

Baldani, M.M., Pascoal, G.B. & Rinaldi, A.E.M. (2018). Rotulagem e promoção comercial de fórmulas infantis comercializadas no Brasil. *Demetra: Alimentação, Nutrição & Saúde*, [S.l.] 13(2), 413-425. doi: 10.12957/demetra.2018.32911

Baranowska, B., Doroszewska, A., Sys, D., Kiersnowska, I., Aleksandrowicz, A., Bernatowicz-Łojko, U., Rabijewski, M., Kajdy, A., & Wesołowska, A. Promotion of Human Milk Substitutes in the Opinion of Mothers of Young Children. *Journal of Food and Nutrition Research*. 2020; 8(7):329-336. doi: 10.12691/jfnr-8-7-4

Barennes, H., Andriatahina, T., Latthaphasavang, V., Anderson, M., & Srour, L. M. (2008). Misperceptions and misuse of Bear Brand coffee creamer as infant food: national cross sectional survey of consumers and paediatricians in Laos. *BMJ* (Clinical research ed.), 337, a1379. https://doi.org/10.1136/bmj.a1379

Barennes, H., Empis, G., Quang, T. D., Sengkhamyong, K., Phasavath, P., Harimanana, A., Sambany, E. M., & Koffi, P. N. (2012). Breast-milk substitutes: a new old-threat for breastfeeding policy in developing countries. A case study in a traditionally high breastfeeding country. *PloS one,* 7(2), e30634. https://doi.org/10.1371/journal.pone.0030634

Barry, S.; Buckle, H.; Newhook, L. A. A.; Twells, L.; Yi, Y.; Roebothan, B. (2020). A preliminary investigation into the potential exposure of mothers to violations of the WHO International Code of Marketing of Breast-Milk Substitutes in Eastern Newfoundland and Labrador, Canada. *Maternal and Child Nutrition*. 16. https://doi.org/10.1111/mcn.12933

Bartolini, F.L.S., Amaral, M., Vilela, Pinto, M.A.P., Mendonça, A.E.d., Vilela, F.M.P., Amaral, L.H.d., & Gomes, F.A. (2009). Official monitoring of the Brazilian Norm for Commercialization of Food for Nursling and Children of First Infancy, Rubber Nipples, Pacifiers, and Nursing Bottles - *NBCAL. Brazilian Journal of Pharmaceutical Science*s 45(3), 475-482. https://doi.org/10.1590/S1984-82502009000300013.

Basch, C. H., Shaffer, E. J., Hammond, R., & Rajan, S. (2013). Prevalence of infant formula advertisements in parenting magazines over a 5-year span. *Journal of pediatric nursing*, 28(6), e28–e32. https://doi.org/10.1016/j.pedn.2013.07.001

Berber, L.C.L. (2016). Educação ou publicidade? Análise dos materiais impressos disponibilizados a profissionais de saúde em um congresso de pediatria [Bachelor in Nutrition Thesis, Universidade de Brasília]. Universidade de Brasília. Respository: https://bdm.unb.br/handle/10483/13922

Berry, N. J., & Gribble, K. D. (2017). Health and nutrition content claims on websites advertising infant formula available in Australia: A content analysis. *Maternal & child nutrition*, 13(4), e12383. https://doi.org/10.1111/mcn.12383

Berry, N. J., Jones, S., & Iverson, D. (2010). It's all formula to me: women's understandings of toddler milk ads. *Breastfeeding Review* 18(1), 21–30.

Berry, N. J.; Jones, S. C.; Iverson, D. (2012). Circumventing the WHO Code? An observational study. *Archives of Disease in Childhood* 97: 320-5. doi: 10.1136/adc.2010.202051

Bertha, J. & Caicedo-Borrás, R. (2016). Estudio Para Estimar la Prevalencia de Violaciones al Codigo Internacional de Comercializacion de Sucedaneos de Leche Materna en dos Ciudades de Ecuador. FUNBBASIC/IBFAN. https://www.salud.gob.ec/wp-content/uploads/2019/07/3_estudio_prevalenciade_violaci%C3%B3n_CICSLM.pdf

Brewer B.K., Andrzejewski, C., Vij, V., Muwakki, L., Lo, A., & Evans, A. (2018). *In-Country Assessments of Baby Food Companies' Compliance with the International Code of Marketing of Breast-milk Substitutes: Nigeria Report*. Westat & Access to Nutrition Fund. https://accesstonutrition.org/app/uploads/2020/02/BMS_Westat-Nigeria_Full_Report_2018.pdf

Brewer B.K., Andrzejewski, C., Vij, V., Muwakki, L., Lo, A., & Evans, A. (2018). *In-Country Assessments of Baby Food Companies' Compliance with the International Code of Marketing of Breast-milk Substitutes: Thailand Report*. Westat & Access to Nutrition Fund. https://accesstonutrition.org/app/uploads/2020/02/FINAL_Thailand_Report_20180204.pdf

Brewer, B.K., Salgado, R.V., Evans, A., Lo, A., & Bhatia, R. (2021). *National Assessment on the Compliance with the Code and the National Measures: Mexico Report*. Westat & Access to Nutrition Fund. https://accesstonutrition.org/app/uploads/2021/06/ATNF_Mexico_Report_Final_06.2021.pdf

Brewer, B.K., Salgado, R.V., Evans, A., Lo, A., & Bhatia, R. (2021). *National Assessment on the Compliance with the Code and the National Measures: Philippines Report*. Westate & Access to Nutrition Fund. https://accesstonutrition.org/app/uploads/2021/05/ATNF_Phillipines_Report_FINAL_05.04.21.pdf

Bridge, G., Lomazzi, M., & Bedi, R. (2020). A cross-country exploratory study to investigate the labelling, energy, carbohydrate and sugar content of formula milk products marketed for infants. *British dental journal*, 228(3), 198–212. https://doi.org/10.1038/s41415-020-1252-0

Bridge, G., Lomazzi, M., Santoso, C., & Bedi, R. (2021). Analysis of the labelling of a sample of commercial foods for infants and young children in 13 countries. *Journal of public health policy*, 42(3), 390–401. https://doi.org/10.1057/s41271-021-00290-1

Britto, L.F., da Silva, A.d.P.V., Mendes, L.G., & Medeiros, S.R.A. (2016). Avaliação da Rotulagem de Alimentos à Base de Cereais Para a Alimentação de Lactentes e Crianças na Primeira Infância [Evaluation of food labeling of cereal food for infants and children in early childhood.] *Demetra: Alimentação, Nutrição & Saúde* 11(1), 111-120. https://doi.org/10.12957/demetra.2016.16543

Brown, A., Jones, S.W., & Evans, E. (2020). *Marketing of infant milk in
the UK: what do parents see and believe?* First Steps Nutrition Trust. https://static1.squarespace.com/static/59f75004f09ca48694070f3b/t/6053645514d0f3072adec94e/1616077909798/Marketing_of_infant_milk_in_the_UK-what_do_parents_see_and_believe_finala.pdf

Caicedo-Borrás, R., Díaz, A., Bertha, J., Silva-Jaramillo, K. M., & Rivas Mariño, G. (2021). Violations of the International Code of Marketing of Breastmilk Substitutes (WHO Code) in two Ecuadorian cities. *Nutrition*, 87-88, 111206. https://doi.org/10.1016/j.nut.2021.111206

Cattaneo, A., Pani, P., Carletti, C., Guidetti, M., Mutti, V., Guidetti, C., Knowles, A., & Follow-on Formula Research Group (2015). Advertisements of follow-on formula and their perception by pregnant women and mothers in Italy. *Archives of disease in childhood*, 100(4), 323–328. https://doi.org/10.1136/archdischild-2014-306996

Cetthakrikul, N., Kunpurk, W., Phulkerd, S., Prakongsai, P., Thammarangsi, T., Thaichida, C. & Thopothai, T. (2014). Milk code violation in Thai hospitals. *Obesity Reviews* 15: 212. T7:S30.21 https://onlinelibrary.wiley.com/doi/epdf/10.1111/obr.12153

Champeny, M., Hou, K., Diop, E. I., Sy Gueye, N. Y., Pries, A. M., Zehner, E., Badham, J., & Huffman, S. L. (2019). Prevalence, duration, and content of television advertisements for breast milk substitutes and commercially produced complementary foods in Phnom Penh, Cambodia and Dakar, Senegal. *Maternal & child nutrition*, 15 Suppl 4(Suppl 4), e12781. https://doi.org/10.1111/mcn.12781

Champeny, M., Pereira, C., Sweet, L., Khin, M., Ndiaye Coly, A., Sy Gueye, N. Y., Adhikary, I., Dhungel, S., Makafu, C., Zehner, E., & Huffman, S. L. (2016). Point-of-sale promotion of breastmilk substitutes and commercially produced complementary foods in Cambodia, Nepal, Senegal and Tanzania. *Maternal & child nutrition*, 12 Suppl 2(Suppl 2), 126–139. https://doi.org/10.1111/mcn.12272

Champeny, M., Pries, A. M., Hou, K., Adhikary, I., Zehner, E., & Huffman, S. L. (2019). Predictors of breast milk substitute feeding among newborns in delivery facilities in urban Cambodia and Nepal. *Maternal & child nutrition*, 15 Suppl 4(Suppl 4), e12754. https://doi.org/10.1111/mcn.12754

Changing Markets Foundation. (2017). *Milking It – How Milk Formula Companies are Putting Profits Before Science.* Changing Markets Foundation. https://changingmarkets.org/wp-content/uploads/2017/10/Milking-it-Final-report-CM.pdf

Changing Markets Foundation. (2018). *Busting the Myth of Science-Based Formula – An Investigation into Nestlé Infant Milk Products and Claims.* Changing Markets Foundation. https://changingmarkets.org/wp-content/uploads/2018/02/BUSTING-THE-MYTH-OF-SCIENCE-BASED-FORMULA.pdf

Chen, Y. C., Chang, J. S., & Gong, Y. T. (2015). A Content Analysis of Infant and Toddler Food Advertisements in Taiwanese Popular Pregnancy and Early Parenting Magazines. *Journal of human lactation* 31(3), 458–466. https://doi.org/10.1177/0890334415576513

China Consumers Association. (2019). *2018 report on the promotion and sales of breast-milk substitute*. China Consumers Association. https://www.cqn.com.cn/pp/content/2019-03/07/content_6859399.htm

Ching, C., Zambrano, P., Nguyen, T. T., Tharaney, M., Zafimanjaka, M. G., & Mathisen, R. (2021). Old Tricks, New Opportunities: How Companies Violate the International Code of Marketing of Breast-Milk Substitutes and Undermine Maternal and Child Health during the COVID-19 Pandemic. *International journal of environmental research and public health*, 18(5), 2381. https://doi.org/10.3390/ijerph18052381

de Oliveira, M., Boccolini, C. S., & Fonseca Sally, E. O. (2021). Breastmilk Substitutes Marketing Violations and Associated Factors in Rio de Janeiro, Brazil. *Journal of human lactatio*n 37(1), 158–168. https://doi.org/10.1177/0890334420978405. For charting, combined with Bertoldo, L.A.A. Análise Da Adequação Da Informação E Da Comunicação Do Marketing De Produtos Concorrentes Do Aleitamento Materno E Da Alimentação Complementar Saudável (Portuguese). Instituto de Comunicação e Informação Científica e Tecnológica em Saúde, Rio de Janeiro, 2020. AND Silva, K. B. D.; Oliveira, M. I. C.; Boccolini, C. S.; Sally, E. O. F. (2020). Illegal commercial promotion of products competing with breastfeeding. Rev Saude Publica. 54: 10. AND Boccolini, C. S. B.; Couto De Oliveira, M. I.; Afonso Bertoldo, L.; Borges Da Silva, K.; Priscila Nunes Rodrigues, G. (2017). Illegal commercial sales of infant formula and infant products in Rio de Janeiro city, Brazil. Annals of Nutrition and Metabolism. 71: 407-408. IUNS Conference

Dearlove, T., Begley, A., Scott, J. A., & Devenish-Coleman, G. (2021). Digital Marketing of Commercial Complementary Foods in Australia: An Analysis of Brand Messaging. *International Journal of Environmental Research and Public Health*, 18(15), 7934. MDPI AG. http://dx.doi.org/10.3390/ijerph18157934

Departamento de Nutrição Ministério da saúde Moçambique. (2017). Monitória Da Implementaҫão Do Código De Comercializaҫão Dos Substitutos Do Leite Materno (SLM) Na Província de Manica. UNICEF.

Departamento de Nutrición,Facultad de Medicina, Universidad de Chile & PAHO. (2017). *Monitoreo al Código Internacional de Comercialización de Sucedáneos de la Leche Materna en Santiago, Chile.* Universidad de Chile & PAHO. https://dipol.minsal.cl/wrdprss_minsal/wp-content/uploads/2018/02/INFORME-FINAL-MONITOREO-CICSLM-EN-CHILE-2017.pdf

Dodgson, J. E., Watkins, A. L., Bond, A. B., Kintaro-Tagaloa, C., Arellano, A., & Allred, P. A. (2014). Compliance with the International Code of Marketing of breast-milk substitutes: an observational study of pediatricians' waiting rooms. *Breastfeeding medicin*e 9(3), 135–141. https://doi.org/10.1089/bfm.2013.0096

Durako S.J., Brewer, B.K., Vij, V., Lo, A., & Stone, K. (2016) *In-Country Assessments of BMS Companies’ Compliance with the International Code of Marketing of Breast-milk Substitutes: India Report.* Westat & Access to Nutrition Foundation. https://accesstonutrition.org/app/uploads/2020/02/BMS_Westat-India_Full_Report_2016.pdf

Durako S.J., Thompson, M., Diallo, M. S., & Aronson K. *In-Country Assessments of BMS Companies’ Compliance with the International Code of Marketing of Breast-milk Substitutes: Indonesia Report*. Westat & Access to Nutrition Foundation. https://accesstonutrition.org/app/uploads/2020/02/BMS_Westat-Indonesia_Full_Report_2016.pdf

Durako S.J., Thompson, M., Diallo, M. S., & Aronson K. *In-Country Assessments of BMS Companies’ Compliance with the International Code of Marketing of Breast-milk Substitutes: Vietnam Report*. Westat & Access to Nutrition Foundation. https://accesstonutrition.org/app/uploads/2020/02/BMS_Westat-Vietnam_Report_2016.pdf

Dusdieker, L. B., Dungy, C. I., & Losch, M. E. (2006). Prenatal office practices regarding infant feeding choices. *Clinical pediatrics*, 45(9), 841–845. https://doi.org/10.1177/0009922806294220

Emerson, J., Kouassi, F., Oka Kouamé, R., Damey, F. N., Cisse, A. S., & Tharaney, M. (2021). Mothers' and health workers' exposure to breastmilk substitutes promotions in Abidjan, Côte d'Ivoire. *Maternal & child nutrition*, 17(4), e13230. https://doi.org/10.1111/mcn.13230

Ergin, A., Hatipoğlu, C., Bozkurt, A. I., Erdoğan, A., Güler, S., Ince, G., Kavurgacı, N., Oz, A., & Yeniay, M. K. (2013). Compliance status of product labels to the international code on marketing of breast milk substitutes. *Maternal and child health journal*, 17(1), 62–67. https://doi.org/10.1007/s10995-012-0971-5

Feeley, A. B., Ndeye Coly, A., Sy Gueye, N. Y., Diop, E. I., Pries, A. M., Champeny, M., Zehner, E. R., & Huffman, S. L. (2016). Promotion and consumption of commercially produced foods among children: situation analysis in an urban setting in Senegal. *Maternal & child nutrition*, 12 Suppl 2(Suppl 2), 64–76. https://doi.org/10.1111/mcn.12304

Foss, K. A., & Southwell, B. G. (2006). Infant feeding and the media: the relationship between Parents' Magazine content and breastfeeding, 1972-2000. *International breastfeeding journal*, 1, 10. https://doi.org/10.1186/1746-4358-1-10

Foster, J. (2014). Hospital distribution of formula sample packs in the state of Ohio. *Breastfeeding Medicine*. 9: S-13. https://doi.org/10.1089/bfm.2014.9976.abstracts

Funduluka, P., Bosomprah, S., Chilengi, R., Mugode, R. H., Bwembya, P. A., & Mudenda, B. (2018). Marketing of breast-milk substitutes in Zambia: evaluation of compliance to the international regulatory code. *Journal of public health* (Oxford, England), 40(1), e1–e7. https://doi.org/10.1093/pubmed/fdx023

García-Flores, E., Herrera-Maldonado, N., Martínez-Peñafiel, L., & Pesqueira- Villegas, E. (2017). Violaciones al Código Internacional de Comercialización de Sucedáneos de Leche Materna en México. *Acta Pediátrica de México*, 38(4), 288-290. doi:http://dx.doi.org/10.18233/APM38No4pp288-2901439

Grummer-Strawn, L. M., Holliday, F., Jungo, K. T., & Rollins, N. (2019). Sponsorship of national and regional professional paediatrics associations by companies that make breast-milk substitutes: evidence from a review of official websites. BMJ open, 9(8), e029035. https://doi.org/10.1136/bmjopen-2019-029035

Gunter, B., Dickinson, R., Matthews, J., & Cole, J. (2013), "Formula manufacturers’ web sites: are they really non‐compliant advertisements?", *Health Education*, Vol. 113 No. 1, pp. 18-27. https://doi.org/10.1108/09654281311293619

Gupta, A. (2021). Under Attack: A report of the monitoring the compliance with the Infant milk substitutes, Feeding bottles and Infant foods (Regulation of Production, Supply and Distribution) Act 1992 and the Amendment Act 2003. Breastfeeding Promotion Network of India (BPNI). https://www.bpni.org/wp-content/uploads/2021/05/Under-Attack-Report-May-2021.pdf

Hadihardjono, D. N., Green, M., Stormer, A., Agustino, Izwardy, D., & Champeny, M. (2019). Promotions of breastmilk substitutes, commercial complementary foods and commercial snack products commonly fed to young children are frequently found in points-of-sale in Bandung City, Indonesia. *Maternal & child nutrition*, 15 Suppl 4(Suppl 4), e12808. https://doi.org/10.1111/mcn.12808

Han, S. (2020). *Marketing of (BMS): Content Analysis of Breast Milk Substitutes Digital Marketing on Chinese e-Commerce Platforms*. [MSc Thesis, Yale School of Public Health]. https://elischolar.library.yale.edu/ysphtdl/1944/

Harris J.L., Fleming-Milici, F., Frazier W., Haraghey, K., Kalnova, S., Romo-Palafox, M., Seymour, N., Rodrigues-Arauz, G., & Schwartz, M.B. (2017). *Baby food FACTS. Nutrition and marketing of baby and toddler food and drink*s. UConn Rudd Center for Food Policy & Obesity.

Hastings, G., Angus, K., Eadie, D., & Hunt, K. (2020). Selling second best: how infant formula marketing works. *Globalization and health,* 16(1), 77. https://doi.org/10.1186/s12992-020-00597-w

Helen Keller International & World Vision International (WVI). (2019). *Point-of-Sale Promotion and Labeling Violations of Breastmilk Substitutes in Cambodia: Observations in Six Provinces. Second Phase, 2018*. Helen Keller & WVI. https://archnutrition.org/wp-content/uploads/sites/2/2019/09/BMS-violation-joint-report_final_Aug2019.pdf

Helen Keller International. (2021). *Background Paper: After Cambodian Children Hospitalized with Severe Iron Deficiency and Anemia, Independent Testing of Nutrilatt Infant Formula Commissioned by the Government of Cambodia Finds Dangerously Low Levels of Iron and Zinc When Compared to Product Label Claims*. Helen Keller International.

Hernández-Cordero, S., Lozada-Tequeanes, A. L., Shamah-Levy, T., Lutter, C., González de Cosío, T., Saturno-Hernández, P., Rivera Dommarco, J., & Grummer-Strawn, L. (2019). Violations of the International Code of Marketing of Breast-milk Substitutes in Mexico. *Maternal & child nutrition*, 15(1), e12682. https://doi.org/10.1111/mcn.12682 Combined with Instituto Nacional de Salud Pública, Universidad Iberoamericana, & UNICEF México. (2020). Prevalencia de violaciones al Código Internacional de Comercialización de Sucedáneos de la Leche Materna en México.

Hickman, N., Morgan, S., Crawley, H., & Kerac, M. (2021). Advertising of Human Milk Substitutes in United Kingdom Healthcare Professional Publications: An Observational Study. *Journal of human lactation*. https://doi.org/10.1177/08903344211018161

Hidayana, I., Februhartanty, J., & Parady, V. A. (2017). Violations of the International Code of Marketing of Breast-milk Substitutes: Indonesia context. *Public health nutrition*, 20(1), 165–173. https://doi.org/10.1017/S1368980016001567

Hou, K., Green, M., Chum, S., Kim, C., Stormer, A., & Mundy, G. (2019). Pilot implementation of a monitoring and enforcement system for the International Code of Marketing of Breast-milk Substitutes in Cambodia. *Maternal & child nutrition*, 15 Suppl 4(Suppl 4), e12795. https://doi.org/10.1111/mcn.12795

Howard, C. R., Howard, F. M., & Weitzman, M. L. (1994). Infant formula distribution and advertising in pregnancy: a hospital survey. *Birth* 21(1), 14–19. https://doi.org/10.1111/j.1523-536x.1994.tb00910.x

Huang, Y., Labiner-Wolfe, J., Huang, H., Choiniere, C. J., & Fein, S. B. (2013). Association of health profession and direct-to-consumer marketing with infant formula choice and switching. *Birth* 40(1), 24–31. https://doi.org/10.1111/birt.12025

IBFAN Asia. (2018). *Report on the Monitoring of the Code in 11 Countries of Asia*. IBFAN. https://www.bpni.org/wp-content/uploads/2018/12/Monitoring-of-the-Code-in-11-Countries-of-Asia.pdf

IBFAN Brasil. (2020). *Resultados del Monitoreo Brasileno del Codigo Internacional de Comercializacion de Sucedaneos de la Leche Materna NBCAL: LEY 11.265 y Decreto 9.579/2018.* IBFAN Brasil. http://www.babymilkaction.org/wp-content/uploads/2021/07/BRASIL-monitoreo-2020-final-espan%CC%83ol.pdf

IBFAN Chile. (2021). *Chile 2021: Reporte del monitoreo del Código Internacional de Comercialización de Sucedáneos de Leche Materna (CICSLM*). IBFAN Chile. http://www.babymilkaction.org/wp-content/uploads/2021/06/Informe-Monitoreo-Chile-2021-final-.pdf

IBFAN Mexico. (2020). *Reporte del monitoreo del Código Internacional de Comercialización de Sucedáneos de Leche Materna* (CICSLM). IBFAN Mexico. https://elpoderdelconsumidor.org/wp-content/uploads/2021/07/d-2107-lactancia-codigo-monitoreo-ibfan-mx-informe.pdf

IBFAN-ICDC. (2017). *Breaking the Rules (BTR), Stretching the Rules 2017: Evidence of violations of the International Code of Marketing of Breastmilk Substitutes and subsequent resolutions,compiled from June 2014 to June 2017*. IBFAN. http://www.babymilkaction.org/wp-content/uploads/2021/04/2017-BTR-2017sm.pdf

Interagency Group on Breastfeeding Monitoring (IGBM), UNICEF UK, Ministry of Health of Uganda, & UNICEF Uganda. (2005). *Monitoring of Compliance with the ‘International Code of Marketing of Breast-milk Substitutes’, subsequent World Health Assembly Resolutions and ‘The Food and Drugs (Marketing of Infant and Young Child Foods) Regulations, 1997 ’ in Kampala, Uganda*: Technical Country Report. UNICEF. https://documentcloud.adobe.com/link/track?uri=urn:aaid:scds:US:d38e49c6-6686-4b4c-a681-0ae2170ed749

Karageuzián, G., Vidal, L., De León, C., Girona, A., & Ares, G. (2021). Marketing of commercial foods for infant and young children in Uruguay: Sugary products, health cues on packages and fun social products on Facebook. *Public Health Nutrition*, 1-13. doi:10.1017/S1368980021002780

Khan & Hamid. (2014). *The "Breastfeeding Code" - Overcoming Implementation Barriers in Pakistan*. WHO Improving Program Implementation through Embedded Research (iPIER) program. http://www.emro.who.int/images/stories/rpc/ipier-14-30-pakistan.pdf?ua=1

Kharade A. (2019). *Impact of Marketing Breastmilk Substitutes on Exclusive Breastfeeding in Curaçao*. [Masters Thesis, Maastricht University]. https://www.researchgate.net/publication/350398774_Impact_of_Marketing_Breastmilk_Substitutes_on_Exclusive_Breastfeeding_in_Curacao

Kitoko, P. M. (1997). *Práticas de Alimentação Infantil em Luanda: Evolução nas últimas décadas, padrões atuais, determinantes do desmame precoce e controle da propaganda de substitutos do leite materno.* [Tese de Doutorado, Faculdade de Saúde Pública, Universidade de São Paulo, São Paulo]. doi:10.11606/T.6.2019.tde-03052019-145527.

Klaus, B., & Gherardini, M. (2014). Italy: Hidden Advertising: Italian Antitrust Authority Fined Economic Operator for Misleading Advertising of a Powdered Milk and Baby Bottle Displayed in a Weekly Magazine. *European Food and Feed Law Review*, 9(2), 130–130. http://www.jstor.org/stable/24326039

Kpaibé A., Kouassi Y., Yao N., N’bra A., Dibi S., & Aké, M. (2019). Fat Content and Fatty Acids Profile in Follow-on Formulas Commercialized in Côte d'Ivoire. *Food Science and Nutrition Studies* 3(2). https://doi.org/10.22158/fsns.v3n2p54

Laillou, A., Gerba, H., Zelalem, M., Moges, D., Abera, W., Chuko, T., . . . Chitekwe, S. (2021). Is the legal framework by itself enough for successful WHO code implementation? A case study from Ethiopia. Maternal & Child Nutrition, 17(1), e13059. https://doi.org/10.1111/mcn.13059

Levitt, C. A., Kaczorowski, J., Hanvey, L., Avard, D., & Chance, G. W. (1996). Breast-feeding policies and practices in Canadian hospitals providing maternity care. *CMAJ: Canadian Medical Association journal*, 155(2), 181–188. https://www.ncbi.nlm.nih.gov/pmc/articles/PMC1487952/

Li, J., Nguyen, T. T., Duan, Y., Mathisen, R., & Yang, Z. (2021). Advice to use infant formula and free samples are common in both urban and rural areas in China: a cross-sectional survey. *Public health nutrition*, 24(8). https://doi.org/10.1017/S1368980020005364

Lima, T.B. (2019). Promoção comercial e rotulagem de bicos, mamadeiras e chupetas: avanços no cumprimento da NBCAL. [Bachelor of Nutrition Thesis, Universidade Federal do Maranhão]. http://hdl.handle.net/123456789/3997

Liu, A., Dai, Y., Xie, X., & Chen, L. (2014). Implementation of international code of marketing breast-milk substitutes in China. *Breastfeeding medicine* 9(9), 467–472. https://doi.org/10.1089/bfm.2014.0053

Lopes, A. G. (2013). *Aplicação sanitária da norma brasileira para comercialização de bicos, mamadeiras e chupetas - NBCAL em estabelecimentos comerciais de Piracicaba* - SP. [Dissertação (mestrado profissional) - Universidade Estadual de Campinas, Faculdade de Odontologia de Piracicaba, Piracicaba, SP]. <http://www.repositorio.unicamp.br/handle/REPOSIP/290341>.

Lozada-Tequeanes, A. L., Hernández-Cordero, S., & Shamah-Levy, T. (2020). Marketing of breast milk substitutes on the internet and television in Mexico. *Journal of paediatrics and child health*, 56(9), 1438–1447. https://doi.org/10.1111/jpc.14968

Maastrup, R., Haiek, L. N., & Neo-BFHI Survey Group (2019). Compliance with the "Baby-friendly Hospital Initiative for Neonatal Wards" in 36 countries. *Maternal & child nutrition*, 15(2), e12690. https://doi.org/10.1111/mcn.12690 Combined with Pavicic Bosnjak, A. (2019). Importance of the baby friendly hospital initiative for neonatal wards (Neo BFHI). Journal of Perinatal Medicine. 47: eA301; Niela-VilÉN, H.; Ezeonodo, A.; Maastrup, R.; Haiek, L. N.; Hannula, L. (2020). Neonataaliosastojen Vauvamyönteisyysohjelman (Neo-BFHI) suositusten toteutuminen vastasyntyneiden teho- ja tarkkailuosastoilla Suomessa. Hoitotiede, Hoitotieteiden Tutkimusseura HTTS r.y. 32: 250-261; Abolyan, L. V.; Haiek, L. N.; Pastbina, I. M.; Maastrup, R. (2021). Compliance With the "Baby-Friendly Hospital Initiative for Neonatal Wards" in Russian Hospitals. J Hum Lact: 8903344211002754.

Martens P. J. (2000). Does breastfeeding education affect nursing staff beliefs, exclusive breastfeeding rates, and Baby-Friendly Hospital Initiative compliance? The experience of a small, rural Canadian hospital. *Journal of human lactation* 16(4), 309–318. https://doi.org/10.1177/089033440001600407

McCann, J. R., Russell, C. G., Campbell, K. J., & Woods, J. L. (2021). Nutrition and packaging characteristics of toddler foods and milks in Australia. *Public health nutrition*, 24(5), 1153–1165. https://doi.org/10.1017/S1368980020004590

McInnes, R., Wright, C., Haq, S., & McGranachan, M. (2007). Who's keeping the code? Compliance with the international code for the marketing of breast-milk substitutes in Greater Glasgow. *Public Health Nutrition*, 10(7), 719-725. doi:10.1017/S1368980007441453

Mehdi T, Rizvi TW. (1998) *Feeding fiasco: pushing commercial infant foods in Pakistan.* The Network Association for Rational Use of Medication in Pakistan.

Merewood, A., Fonrose, R., Singleton, M., Grossman, X., Navidi, T., Cook, J.T., & Pomales, T. From Maine to Mississippi: hospital distribution of formula sample packs along the Eastern Seaboard. *Archives of Pediatrics & Adolescent Medicine*. 162: 823-7.DOI: 10.1001/archpedi.162.9.823

Merewood, A., Grossman, X., Cook, J., Sadacharan, R., Singleton, M., Peters, K., & Navidi, T. (2010). US hospitals violate WHO policy on the distribution of formula sample packs: results of a national survey. *Journal of human lactation* 26(4), 363–367. https://doi.org/10.1177/0890334410376947

Mialon, M., Jaramillo, Á., Caro, P., Flores, M., González, L., Gutierrez-Gómez, Y., Lay, L., López-Arana, S., López-Bautista, F., Mata, C., Moliterno, P., Palomares, L., Páramo, K., Rauber, F., & Rivas-Mariño, G. (2021). Involvement of the food industry in nutrition conferences in Latin America and the Caribbean. *Public health nutrition*, 24(6), 1559–1565. https://doi.org/10.1017/S1368980020003870

Ministerio de Salud del Perú, UNICEF, & PAHO. (2011). *La lactancia materna y el cumplimiento del código internacional de comercialización de sucedáneos de leche materna en el Perú. Informe final de monitoreo en Apurímac, Ayacucho, Huancavelica, Lima y Loreto.* PAHO. https://cdn.www.gob.pe/uploads/document/file/391013/La_lactancia_materna_y_el_cumplimiento_del_c%C3%B3digo_internacional_de_comercializaci%C3%B3n_de_suced%C3%A1neos_de_leche_materna_en_el_Per%C3%BA._Informe_final_de_monitoreo_en_Apur%C3%ADmac__Ayacucho__Huancavelica__Lima_y_Loreto20191017-26355-1i1w4mb.pdf

Ministerio de Salud Publica & IBFAN (2012). *Vigilancia del cumplimiento del codigo internacional de comercializacion de sucedeaneos de la leche materna en Ecuador*. Ministerio de Salud Publica & IBFAN. http://www.ibfan-alc.org/noticias/Monitoreo-Ecuador-2011.pdf

Ministerio de Salud, UNICEF, & PAHO. (2019). Uruguay Monitoreo del cumplimiento del código de comercialización de sucedáneos de la leche materna en Uruguay. PAHO. https://iris.paho.org/bitstream/handle/10665.2/53932/9789974860230_spa.pdf?sequence=1&isAllowed=y

Ministria e Shëndetësisë, UNICEF & IBFAN. (2014). *Albania Raporti I Monitorimittë Praktikave Të Tregtimittë Zëvendësuesve Të Qumështit Të Gjirit Në Shqipë*r (Monitoring Report on Trading Practices of Breast Milk Substitutes in Albania). Ministria e Shëndetësisë.

Morgan, S., Waterston, T., & Kerac, M. (2018). Infant formula advertising in medical journals: a cross-sectional study (and struggle to publish). *Field Exchange.* Emergency Nutrition Network (ENN): 29-30. https://www.ennonline.net/attachments/2919/FEX_58_BMS_ads_p29.pdf and Morgan, S.; Waterston, T.; Kerac, M. (2014). How common are infant formula advertisements in leading medical journals and do they risk subverting breastfeeding? Archives of Disease in Childhood. 99: A177-A178.

Mugure, F.T. (2013). *Assessing Compliance with the International Code of Marketing of Breastmilk Substitutes: Report of An Assessment in Three Counties in Kenya*. Ministry of Health Kenya, UNICEF, & PATH.

Muravha, N. (2014). Violations of the International Code of Marketing of Breast Milk Substitutes in South African health facilities. [Masters Thesis, North-West University]. https://repository.nwu.ac.za/handle/10394/15459

Nguyen, T.S., Barraclough, S., Morrow, M., & Duong Quang, T. (2000). Controlling infant formula promotion in Ho Chi Minh City, Vietnam: Barriers to policy implementation in the health sector. *Australian Journal of Primary Health - Interchange*. 6: 27-36.

Oliveira, I.M., & Camozzi, A.B.Q. (2021). *Inadequação na promoção comercial de alimentos para lactentes segundo a NBCA*L. [Thesis, Pontifical Catholic University of Goiás]. https://repositorio.pucgoias.edu.br/jspui/bitstream/123456789/1781/1/Inadequa%C3%A7%C3%A3o%20na%20promo%C3%A7%C3%A3o%20comercial%20de%20alimentos%20para%20lactentes%20segundo%20a%20NBCAL.pdf

PADILHA, F. (2011). *Rotulagem e propaganda comercial de fórmulas infantis para lactentes e crianças de primeira infância*. [Dissertação (Mestrado), Programa de Pós-Graduação em Nutrição, Universidade Federal de Pernambuco]. https://repositorio.ufpe.br/handle/123456789/8305

Pagnoncelli, M., Mariz Batista, A., Silva, M., Costa, A., Araújo, F., Marques, M., Fidalgo, C., & Carvalho, M. (2009). Analysis of advertisements of infant food commercialized in the city of Natal, Rio Grande do Norte, Brazil. *Brazilian Journal of Pharmaceutical Sciences - BRAZ J PHARM SCI*, 45. https://doi.org/10.1590/S1984-82502009000200020

Parady, U.; Februhartanty, J.; Blaney, S.; Sukotjo, S. (2013). Monitoring the compliance to the international Code of Marketing of Breastmilk Substitutes: Labelling violations in Indonesia. *Annals of Nutrition and Metabolism*. 63: 494-494.

Parrilla-Rodríguez, A. M., & Gorrín-Peralta, J. J. (2008). Formula labeling violations to the WHO Code: a quantitative and qualitative analysis. *Puerto Rico health sciences journal*, 27(1), 49–54.

Paula, L. O., Chagas, L. R., & Ramos, C. V. (2010). Monitoramento da norma brasileira de comercialização de alimentos infantis. *Nutrire: J. Brazilian Soc. Food Nutr*. (35) 3, 43-55.

Pomeranz, J. L., Romo Palafox, M. J., & Harris, J. L. (2018). Toddler drinks, formulas, and milks: Labeling practices and policy implications. *Preventive medicine*, 109, 11–16. https://doi.org/10.1016/j.ypmed.2018.01.009

Popkin, B. M., Fernandez, M. E., & Avila, J. L. (1990). Infant formula promotion and the health sector in the Philippines. *American journal of public health*, 80(1), 74–75. https://doi.org/10.2105/ajph.80.1.74

Prado, I., & Rinaldi, A. (2020). Compliance of infant formula promotion on websites of Brazilian manufacturers and drugstores. *Revista de saude publica*, 54, 12. https://doi.org/10.11606/s1518-8787.2020054001327

Pries, A. M., Huffman, S. L., Adhikary, I., Upreti, S. R., Dhungel, S., Champeny, M., & Zehner, E. (2016). High consumption of commercial food products among children less than 24 months of age and product promotion in Kathmandu Valley, Nepal. *Maternal & child nutrition*, 12 Suppl 2(Suppl 2), 22–37. https://doi.org/10.1111/mcn.12267

Pries, A. M., Huffman, S. L., Adhikary, I., Upreti, S. R., Dhungel, S., Champeny, M., & Zehner, E. (2016). Promotion and prelacteal feeding of breastmilk substitutes among mothers in Kathmandu Valley, Nepal. *Maternal & child nutrition*, 12 Suppl 2(Suppl 2), 8–21. https://doi.org/10.1111/mcn.12205

Pries, A. M., Huffman, S. L., Mengkheang, K., Kroeun, H., Champeny, M., Roberts, M., & Zehner, E. (2016). Pervasive promotion of breastmilk substitutes in Phnom Penh, Cambodia, and high usage by mothers for infant and young child feeding. *Maternal & child nutrition*, 12 Suppl 2(Suppl 2), 38–51. https://doi.org/10.1111/mcn.12271

Pries, A. M., Mulder, A., Badham, J., Sweet, L., Yuen, K., & Zehner, E. (2021). Sugar content and nutrient content claims of growing-up milks in Indonesia. *Maternal & child nutrition*, 17(4), e13186. https://doi.org/10.1111/mcn.13186

Radebe, P. (2014). *Assessing the extent of violations of the International Code of Marketing of Breast Milk Substitutes in South African advertising medi*a. [Masters Thesis, North-West University]. https://repository.nwu.ac.za/handle/10394/15517

Rea, M. F., & Toma, T. S. (2000). Proteção do leite materno e ética [Protection of mother's milk and ethics]. *Revista de saude publica*, 34(4), 388–395. https://doi.org/10.1590/s0034-89102000000400012

Rodrigues, G. P. N., Oliveira, M. I. C., Boccolini, C. S., Sally, E. O. F., & Moraes, J. R. (2021). [Impact assessment of an educational intervention in pharmacies that use commercial promotion of products competing with maternal breastfeeding]. *Cad Saude Publica*. 37: e00129919. https://doi.org/10.1590/0102-311X00129919

Rothstein, J. D., Caulfield, L. E., Broaddus-Shea, E. T., Muschelli, J., Gilman, R. H., & Winch, P. J. (2020). "The doctor said formula would help me": Health sector influences on use of infant formula in peri-urban Lima, Peru. *Social science & medicine* (1982), 244, 112324. https://doi.org/10.1016/j.socscimed.2019.05.029

Sadacharan, R., Grossman, X., Sanchez, E., & Merewood, A. (2011). Trends in US hospital distribution of industry-sponsored infant formula sample packs. *Pediatrics*, 128(4), 702–705. https://doi.org/10.1542/peds.2011-0983

Salasibew, M., Kiani, A., Faragher, B., & Garner, P. (2008). Awareness and reported violations of the WHO International Code and Pakistan's national breastfeeding legislation; a descriptive cross-sectional survey. *International Breastfeed Journal* 3(24). https://doi.org/10.1186/1746-4358-3-24

Salve, J. M., Divitiis, R. M. & Toma, T. S. (2008). *Violando as normas 2008: relatório nacional das violações à Norma Brasileira de Comercialização de Alimentos para Lactentes e crianças de primeira infância, bicos, chupetas e mamadeiras e Lei 11.265/06: edição comemorativa dos 20 anos da NBCAL: 138-138*. IBFAN Brasil. http://www.ibfan.org.br/monitoramento/pdf/doc-360.pdf

Sanchez, J. (2013). *Cumplimiento del código internacional de comercialización de sucedáneos de la leche materna y modalidad de consumo de fórmulas infantiles.* (Compliance with the international code of commercialization of breast milk substitutes and mode of consumption of infant formulas). [Bachelor's Thesis: Nutrition FASTA University, Argentina}. http://redi.ufasta.edu.ar

Sanyoto-Besar, D. (2004). *Indonesia code violations: a survey of the state of the International Code of Marketing of Breastmilk Substitutes and subsequent WHA Resolutions* - Pamphlet. IBFAN-ICDC. https://ibfan.org/art/298-4.pdf

Senkal, E., & Yildiz, S. (2019). Violation of the international code of marketing of breastfeeding substitutes (WHO Code) by the formula companies via social media. *Archives of Disease in Childhood*, 104(Suppl 2), A143-A143. doi:10.1136/archdischild-2019-rcpch.338

Shaikh, U., & Scott, B. J. (2005). Extent, Accuracy, and Credibility of Breastfeeding Information on the Internet. *Journal of Human Lactation*, 21(2), 175–183. https://doi.org/10.1177/0890334405275824

Shaker-Berbari, L., Ghattas, H., Symon, A. G., & Anderson, A. S. (2018). Infant and young child feeding in emergencies: Organisational policies and activities during the refugee crisis in Lebanon. *Maternal & child nutrition*, 14(3), e12576. https://doi.org/10.1111/mcn.12576

Sheehan, D., Bridle, B., Hillier, T., Feightner, K., Hayward, S., Lee, K. S., Krueger, P., Sword, W., & James, M. (1999). Breastfeeding outcomes of women following uncomplicated birth in Hamilton-Wentworth. *Canadian journal of public health* 90(6), 408–411. https://doi.org/10.1007/BF03404147

Smith, J., & Blake, M. (2013). Infant food marketing strategies undermine effective regulation of breast-milk substitutes: trends in print advertising in Australia, 1950-2010. *Australian and New Zealand journal of public health*, 37(4), 337–344. https://doi.org/10.1111/1753-6405.12081

Sobel, H. L., Iellamo, A., Raya, R. R., Padilla, A. A., Olivé, J. M., & Nyunt-U, S. (2011). Is unimpeded marketing for breast milk substitutes responsible for the decline in breastfeeding in the Philippines? An exploratory survey and focus group analysis. *Social science & medicine* 73(10), 1445–1448. https://doi.org/10.1016/j.socscimed.2011.08.029

Sokol, E., Thiagarajab, S., & Allain, A. (1998). *Breaking the rules, stretching the rules 1998: a worldwide report on violations of the WHO/UNICEF international code of marketing of breastmilk substitutes. IBFAN-ICDC.*

Stang, J., Hoss, K., & Story, M. (2010). Health Statements Made in Infant Formula Advertisements in Pregnancy and Early Parenting Magazines: A Content Analysis. *ICAN: Infant, Child, & Adolescent Nutrition*, 2(1), 16–25. https://doi.org/10.1177/1941406409359806

Sweet, L., Jerling, J., & Van Graan, A. (2013). Field-testing of guidance on the appropriate labelling of processed complementary foods for infants and young children in South Africa. *Maternal & child nutrition*, 9 Suppl 1(Suppl 1), 12–34. https://doi.org/10.1111/mcn.12019

Sweet, L., Pereira, C., Ford, R., Feeley, A. B., Badham, J., Mengkheang, K., Adhikary, I., Sy Gueye, N. Y., Coly, A. N., Makafu, C., & Zehner, E. (2016). Assessment of corporate compliance with guidance and regulations on labels of commercially produced complementary foods sold in Cambodia, Nepal, Senegal and Tanzania. *Maternal & child nutrition*, 12 Suppl 2(Suppl 2), 106–125. https://doi.org/10.1111/mcn.12268

Taylor A. (1998). Violations of the international code of marketing of breast milk substitutes: prevalence in four countries. *BMJ* (Clinical research ed.), 316(7138), 1117–1122. https://doi.org/10.1136/bmj.316.7138.1117

Toma, T. S. & Rea, M. F. (1997). Rótulos de alimentos infantis: alguns aspectos das práticas de marketing no Brasil. *Rev. nutr. PUCCAMP*. 10: 127-35. https://doi.org/10.1590/S1415-52731997000200006

UNICEF & IBFAN Uganda. (2011). Strengthening Enforcement and Monitoring of Activities on the Regulations on Marketing of Infant Foods. UNICEF & IBFAN Uganda.

Valaitis, R. K., & Shea, E. (1993). An evaluation of breastfeeding promotion literature: does it really promote breastfeeding?. *Canadian journal of public health* 84(1), 24–27.

Vallone, F. (2009). Small great customers. The advertising of breastmilk substitutes in two Argentinean pediatric journals between 1977 and 2006. *Salud Colectiva*, Universidad Nacional de Lanos. 5: 87-105.

Vinje, K. H., Phan, L., Nguyen, T. T., Henjum, S., Ribe, L. O., & Mathisen, R. (2017). Media audit reveals inappropriate promotion of products under the scope of the International Code of Marketing of Breast-milk Substitutes in South-East Asia. *Public health nutrition*, 20(8), 1333–1342. https://doi.org/10.1017/S1368980016003591

Vitta, B. S., Benjamin, M., Pries, A. M., Champeny, M., Zehner, E., & Huffman, S. L. (2016). Infant and young child feeding practices among children under 2 years of age and maternal exposure to infant and young child feeding messages and promotions in Dar es Salaam, Tanzania. *Maternal & child nutrition*, 12 Suppl 2(Suppl 2), 77–90. https://doi.org/10.1111/mcn.12292

Wallace, L. S., Rosenstein, P. F., & Gal, N. (2016). Readability and Content Characteristics of Powdered Infant Formula Instructions in the United States. *Maternal and child health journal*, 20(4), 889–894. https://doi.org/10.1007/s10995-015-1877-9

WHO Regional Office for Europe. (2019). *Commercial foods for infants and young children in the WHO European Region: a study of the availability, composition and marketing of baby foods in four European countries*. WHO. https://www.euro.who.int/en/health-topics/disease-prevention/nutrition/publications/2019/commercial-foods-for-infants-and-young-children-in-the-who-european-region-2019

WHO Regional Office for Europe. (2021). *Commercial foods for infants and young children in Poland*. WHO. https://www.euro.who.int/en/countries/poland/publications/commercial-foods-for-infants-and-young-children-in-poland-2021

Yeong J. K., & Allain, A. (2001). *Breaking the rules, stretching the rules 2001: evidence of violations of the international code of marketing of breastmilk substitutes and subsequent resolutions.* IBFAN-ICDC.

Yeong J K., & Allain, A. (2004). *Breaking the rules, stretching the rules 2004: evidence of violations of the International Code of Marketing of Breastmilk Substitutes and subsequent resolutions.* IBFAN-ICDC. http://ibfan.org/art/302-2.pdf

Zhang, S., Yaohua, D., Liu, J. et al. (2004). Implementation of the Code of Practice for the Sale of Breastmilk Substitutes in Six Cities. *China Journal of Reproductive Health* (04): 203-205.

Zhao, J., Li, M., & Freeman, B. (2019). A Baby Formula Designed for Chinese Babies: Content Analysis of Milk Formula Advertisements on Chinese Parenting Apps. *JMIR mHealth and uHealth*, 7(11), e14219. https://doi.org/10.2196/14219
