# Supplementary material for: Global evidence of persistent violations of the International Code of Marketing of Breast‐milk Substitutes: A systematic scoping review
Source: Matern Child Nutr. 2022 Mar 21;18(Suppl 3):e13335. doi: 10.1111/mcn.13335 (PMC9113471; doi:10.1111/mcn.13335)
Supplement: Supplementary file 3 — Supporting information. [file MCN-18-e13335-s002.docx]

**Supplementary Material A:** Search strategy, Databases and organisation websites searched

| Database | Date Searched | # Search results | # Duplicates removed | De-Duplicated Total |
| --- | --- | --- | --- | --- |
| ProQuest Dissertations and Theses Global | 27/07/2021 | 14 | 1 | 13 |
| PubMed | 27/07/2021 | 135 | 0 | 135 |
| Scopus | 28/07/2021 | 215 | 114 | 101 |
| Web of Science (Core Collection) | 28/07/2021 | 178 | 109 | 69 |
| Global Health | 03/08/2021 | 111 | 88 | 23 |
| Academic Search Premier | 03/08/2021 | 88 | 69 | 19 |
| CINAHL | 03/08/2021 | 78 | 75 | 3 |
| Africa-Wide Information | 03/08/2021 | 27 | 24 | 3 |
| Business Source Premier | 03/08/2021 | 14 | 11 | 3 |
| APA PsycInfo | 03/08/2021 | 14 | 13 | 1 |
| EconLit | 03/08/2021 | 4 | 3 | 1 |
| ERIC | 03/08/2021 | 3 | 1 | 2 |
| Bibliography of Asian Studies | 03/08/2021 | 0 | 0 | 0 |
| Sociological Abstracts | 04/08/2021 | 8 | 4 | 4 |
| Embase | 05/08/2021 | 157 | 115 | 42 |
| SciELO | 06/08/2021 | 14 | 5 | 9 |
| Global Index Medicus | 06/08/2021 | 37 | 12 | 25 |
| CNKI | 17/08/2021 | 14 | 0 | 14 |
| Totals |  | 1111 | 644 | 467 |

**Search terms example of PubMed** (search run on 7/27/2021)

(code[tiab] OR code[ot]) AND ("infant formula"[Mesh] OR "infant food"[Mesh] OR "milk substitutes"[Mesh] OR "milk, human"[Mesh] OR "bottle feeding"[Mesh] OR "breast feeding"[Mesh] OR "child nutritional physiological phenomena"[Mesh] OR "infant nutritional physiological phenomena"[Mesh] OR "maternal nutritional physiological phenomena"[Mesh] OR breast-milk[tiab] OR breastmilk[tiab] OR "breast milk"[tiab] OR "milk substitute*"[tiab] OR "bottle feed*"[tiab] OR "bottle fed"[tiab] OR bottlefeed*[tiab] OR bottlefed[tiab] OR "breast feed*"[tiab] OR breastfeed*[tiab] OR breastfed[tiab] OR "breast fed"[tiab] OR "artificial feeding"[tiab] OR "complementary feeding*"[tiab] OR "complementary food*"[tiab] OR "supplemental feeding*"[tiab] OR "supplementary feeding*"[tiab] OR "follow-up formula"[tiab] OR "follow-on milk"[tiab] OR "follow-on formula"[tiab] OR "growing-up milk"[tiab] OR "growing up milk"[tiab] OR "human milk"[tiab] OR "feeding bottle"[tiab] OR "feeding nipple"[tiab] OR "feeding teat"[tiab] OR "specialized formula"[tiab] OR "condensed milk"[tiab] OR "powdered milk"[tiab] OR "milk powder"[tiab] OR ((newborn*[tiab] OR infant*[tiab] OR baby[tiab] OR babies[tiab] OR toddler*[tiab] OR "young child*"[tiab] OR preschool[tiab] OR pre-school[tiab] OR maternal[tiab] OR mother*[tiab]) AND (formula*[tiab] OR milk*[tiab] OR food*[tiab] OR nutrition[tiab] OR feeding[tiab] OR beverage*[tiab]))) AND (violat*[tiab] OR complian*[tiab] OR non-complian*[tiab] OR inappropriate*[tiab] OR "conflict of interest"[tiab] OR "conflicts of interest"[tiab] OR breach*[tiab] OR aggressive[tiab] OR undermin*[tiab] OR claim*[tiab] OR donation*[tiab] OR sponsorship*[tiab] OR tactic*[tiab] OR cross-promot*[tiab] OR "cross promot*"[tiab])

Filters: 1/1/1981-7/15/2021 Search terms were adapted as necessary to correspond to database formatting.

***Other resources searched***

Web sites of organisations identified as closely involved with the Code were searched using the terms: code, marketing, formula, breast milk substitutes, feeding, breastfeeding, milk, child nutrition, through the index of publications (if available) and the website search tool.

| **Web site** | **Date(s) searched** | **Retrieved for Screening** |
| --- | --- | --- |
| UNICEF <https://www.unicef.org/research-and-reports> <https://data.unicef.org/> | 20 July 2021 | 3 |
| International Baby Food Action Network (IBFAN)/ICDC, via Baby Milk Action UK because the IBFAN site was not functioning <http://www.babymilkaction.org/monitoring> | 20 July to August 26, 2021, | 30 |
| Access to Nutrition Initiative (ATNI), <https://accesstonutrition.org/> | 15 July 2021 | 10 |
| Helen Keller International, <https://www.hki.org/> with particular focus on their ARCH projects <https://archnutrition.org/> | 15 July 2021 | 20 |
| FHI 360/Alive & Thrive, <https://www.aliveandthrive.org/> | 19 to 24 July 2021 | 14 |
| World Vision <https://www.wvi.org/publications> | 19 July 2021 | 2 |
| Save the Children <https://www.savethechildren.net/research-reports> | 30 June 2021 | 4 |
| WHO, <https://www.who.int/publications> then regional sites | 26 August to 20 Sept 2021 | 16 |
| Total |  | 99 |
