# Supplementary material for: Global evidence of persistent violations of the International Code of Marketing of Breast‐milk Substitutes: A systematic scoping review
Source: Matern Child Nutr. 2022 Mar 21;18(Suppl 3):e13335. doi: 10.1111/mcn.13335 (PMC9113471; doi:10.1111/mcn.13335)
Supplement: Supplementary file 4 — Supporting information. [file MCN-18-e13335-s001.docx]

**Supporting Materials B: List of countries where studies reported Code violations**

Some items collected data from multi-countries thus there are more countries than the number of studies.

Albania Angola Argentina Australia

Austria Bangladesh Belgium Benin

Bhutan Bolivia Brazil Bulgaria

Burkina Faso Cambodia Canada Chile

China Columbia Costa Rica Cote d'Ivoire

Croatia Curaçao Denmark Dominican Republic

Ecuador Estonia Ethiopia Finland

France Gabon Gambia Germany

Ghana Guatemala Hungary Iceland

India Indonesia Israel Italy

Japan Kenya Kuwait Laos

Latvia Lebanon Lithuania Luxembourg

Malaysia Maldives Mauritius Mexico

Mongolia Mozambique Myanmar Nepal

New Zealand Nicaragua Niger Nigeria

Norway Oman Pakistan Panama

Paraguay Peru Philippines Poland

Portugal Puerto Rico Republic of Korea Romania

Russia Senegal Singapore Slovenia

South Africa Spain Sri Lanka Sudan

Sweden Switzerland Tanzania Thailand

Togo Turkey UAE Uganda

UK Uruguay USA Venezuela

Vietnam Western Samoa Zambia

*Region named not country*

“Africa, Americas, Asia, Europe, Oceania”

20 countries of Latin America and Caribbean

Europe

North America

*Other*

Health professionals at a pediatric congress in Brazil (but may be from various countries)

14 "global markets"

79 countries in Africa, Asia Pacific, Caribbean, Europe, Latin America, Middle East, North America

40 countries and “all regions of the world”
